# Assessment of the efficacy of an antimicrobial peptide in the context of cystic fibrosis airways

**DOI:** 10.1016/j.crmicr.2025.100367

**Published:** 2025-02-28

**Authors:** Albane Jouault, Inès Jeguirim, Inès Ben Hadj Kaddour, Lhousseine Touqui

**Affiliations:** a Institut Pasteur, Université de Paris Cité, Mucoviscidose et Bronchopathies Chroniques, Paris, France; b Centre de Recherche Saint-Antoine, Sorbonne Université, Inserm, Paris, France

**Keywords:** Antimicrobial peptide, MRSA, Cystic fibrosis, Sputum, Biofilm

## Abstract

Antimicrobial peptides (AMPs) offer a promising alternative to control airway infections with multi-resistant bacteria, such as methicillin-resistant *Staphylococcus aureus* (MRSA), which commonly infects patients with cystic fibrosis (CF). However, the behavior of AMPs in the CF context has yet to be fully elucidated. CF airways produce large amounts of proteases and viscous mucus (sputum), which may affect the efficacy of AMPs. The present work aimed to determine whether CF conditions affect the bactericidal efficacy of CAMA, a promising AMP known to kill clinical MRSA strains efficiently. Using a killing assay, we quantified CAMA bactericidal activity on a CF clinical MRSA strain in the presence of several compounds of CF airways, including sputum and bronchial epithelial cells (BECs). Our results indicate that CF sputum impairs the bactericidal efficacy of CAMA. Similar results were observed when CAMA was incubated with an artificial sputum medium (ASM). When used separately, sputum components (DNA, lipids, and mucins) reproduced the inhibitory effects of ASM. Additionally, the bactericidal efficacy of CAMA was also slightly altered when planktonic *S. aureus* strains were co-cultured with CF BECs. However, CAMA was not active against *S. aureus* cultured on BEC in biofilm mode, characteristic of chronic infections in CF patients. These findings suggest that although CAMA represents a promising tool to treat MRSA strains, the CF environment may impair the efficacy of this AMP. Identifying strategies to protect AMPs from the deleterious effects of CF sputum is a key priority.

## Graphical abstract

**Figure:**
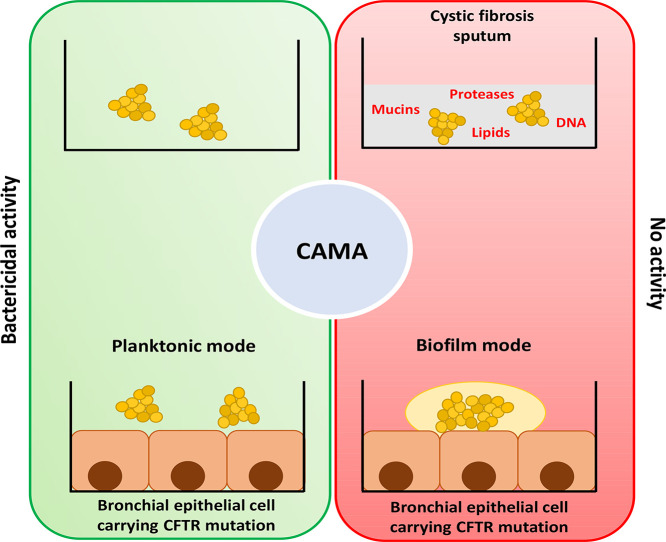

## 1 Introduction

Cystic fibrosis (CF) is an autosomal recessive inherited disorder. This is caused by mutations in the cystic fibrosis transmembrane conductance regulator (CFTR) gene. CFTR dysfunction leads to an imbalance of ions in the airway lumen, resulting in mucus dehydration. This ultimately compromises mucociliary clearance, favoring colonization and infection by opportunistic pathogens (Boucher, 2002). This environment promotes the formation of biofilms, which can result in chronic infections. These infections are the primary causes of morbidity and mortality among CF patients and do not resolve with antibiotic treatment. It is estimated that over 80 % of CF patients will ultimately succumb to respiratory failure caused by bacterial infections and airway inflammation (Lyczak et al., 2002).

Methicillin-resistant *Staphylococcus aureus* (MRSA) is one of the bacteria involved in these infections in CF patients. Its prevalence increased in the early 2000s and currently stands at 18% (CFF patient registry, 2021). There is a clear link between chronic MRSA infection and adverse clinical outcomes, including accelerated decline in lung function, increased hospital admissions and earlier mortality (Blanchard and Waters, 2019, Lu et al., 2023). Chronic MRSA infections continue to pose significant treatment challenges for CF patients. The ability of MRSA to endure high concentrations of bactericidal antibiotics is a primary reason for treatment failures and the necessity for new drugs (Turner et al., 2019).

Antimicrobial peptides (AMPs) represent a promising alternative treatment for MRSA. AMPs are broad-spectrum antimicrobials that rapidly neutralize multiple pathogens including bacteria, yeast, viruses, and fungi (Bugli et al., 2022). They are also defined as host defense peptides with immunomodulatory functions, wound healing, and many other actions on host cells (de la Fuente-Núñez et al., 2017). AMPs are evolutionarily conserved in the genome and produced by most living organisms, playing an essential role in innate immune responses. CAMA is a small hybrid peptide composed of two parts of two different antimicrobial peptides: the N-terminal alpha-helical segment of cecropin A(amino acid 1–7) and melittin A(2–9). It has demonstrated high bactericidal activity against bacterial species that infect the airways of CF patients, particularly against MRSA clinical strains (Geitani et al., 2019). CAMA is also effective against clinical strains of *Pseudomonas aeruginosa*, another pathogen presenting a significant challenge for CF patients (Geitani et al., 2019). When these patients are co-infected with chronic MRSA and *P. aeruginosa*, this leads to an increased decline in their lung function (Maliniak et al., 2016). The use of CAMA could therefore be an effective strategy for combating these two pathogens simultaneously. Given that the bactericidal activity of AMPs is, in large part, based on their electrostatic interactions with targeted bacteria, the high positive charge of CAMA (+8) confers potent antibacterial activity to this AMP against various bacterial species (Geitani et al., 2022). Thus, CAMA is a promising *in vitro* candidate for combating MRSA infections in CF patients.

The characteristics of the site of infection are key factors that can influence the activity of AMPs. The majority of published studies on AMPs including CAMA have employed an *in vitro* approach using host cell-free media to examine the bactericidal effects of AMPs. These conditions do not accurately represent the CF airways context, where injected AMPs come into contact with multiple airway components, including bronchial epithelial cells and viscous mucus. It is worth noting that the airways mucus of patients with CF disease is rich in proteases, lipids, mucins, and DNA, which could potentially degrade or inactivate CAMA (Leal et al., 2017; Niv et al., 2021; Piva et al., 2020; Sahu and Lynn, 1978). Furthermore, the CF airway may also favor biofilm formation, which can lead to chronic bacterial infections that are difficult to treat (Jean-Pierre et al., 2022). The biofilm matrix and the different metabolic states of the bacteria present in the biofilm result in increased tolerance to antibiotics, making treatment more difficult (Ciofu et al., 2022).

To align as closely as possible with the CF context, the present studies investigated whether CAMA maintains its bactericidal activity against *S. aureus* strains in the presence of CF sputum or CF bronchial epithelial cells.

## 2 Materials and methods

### 2.1 Antimicrobial peptide

CAMA (KWKLFKKIGAVLKVL-NH₂, purity > 95%) was purchased from Bachem AG (Bubendorf, Switzerland). The peptide powder was dissolved in sterile water at a 1 mg/mL concentration and stored at −20 °C until use.

### 2.2 Cells and bacterial cultures

The F508del-16HBE bronchial epithelial (CF) cell line (from Sigma-Aldrich®) was maintained in MEM + GlutaMAX™ (Gibco) supplemented with 10% (v/v) Fetal Bovine Serum (FBS; Eurobioscientific), penicillin-streptomycin, (100 U/ml, Gibco) in T175 culture flasks (TPP®) at 37 °C/5% CO_2_ atmosphere.

The CF clinical strain of methicillin-resistant *S. aureus* (MRSA 7877) was provided by Dr. Philippe Morand (Service de Bactériologie at Hôpital Cochin in Paris, France). All strains were stored at −80 °C in 25 % glycerol until use. Bacteria were cultured on an LB agar plate or LB (thermofischer) at 37 °C with shaking (120 rpm). For SA113-GFP, kanamycin at 100 µg/ml was added to the media.

### 2.3 CF sputum collection and treatments

The CF sputum samples were collected from CF patients following the guidelines set forth by the European Union and the local Committees on Human Research CPP Ile-de-France 1. They were registered under the reference number 2013-Nov.-13406. All CF patients provided informed consent. In accordance with our privacy policy, we did not disclose any personal information about the patients. The collected sputa were diluted in phosphate-buffered saline (PBS) (1:1 vol) and then homogenized with repeated vigorous agitation. Aliquots (200 μl) of mixed suspension of sputa were treated with recombinant bovine DNase (10 units, Roche) for 1 h at 37  °C with up–and-down rotation. This process reduces the viscosity of CF sputa, which is caused by an excess of DNA and F-actin in the patient's expectorations (Tang et al., 2005). Then, the supernatants were collected by centrifugation (14,000 *g* at 4  °C for 10 min) and further irradiated in dry ice using a ^137^Cs-source γ-irradiator (IBL 637, CIS bio international) to eliminate any residual bacteria present in the sputa. This was confirmed by streaking an aliquot of the sample on Luria Bertani (LB) -agar plate and checking for bacterial colonies after 48 h of incubation at 37 °C. The samples were subsequently stored at −80  °C for further analysis (Pernet et al., 2014). Before each experiment, a 1:20 dilution of CF sputum was prepared in PBS. To inactivate proteases, a pre-treatment with a protease inhibitor cocktail (Thermofischer) was conducted for 30 min at 37 °C.

### 2.4 Preparation of artificial sputum medium

An artificial sputum medium (ASM) designed to mimic CF mucus was reproduced as previously described using 5 g/L mucins from pig stomach (sigma), 4 g/L salmon sperm DNA, 5.9 mg/L DTPA (sigma), 5 g/L NaCl (sigma), 2.2 g/L KCL(sigma), 1.81 g/L Tris base (sigma), 0.5% (v/v) egg yolk emulsion (lipids) (thermofischer) and 5 g/L casamino acids (sigma) (Sriramulu et al., 2005). To examine the relative role of each component in the whole effect of ASM on CAMA, we prepared different ASM solutions without DNA, mucin or lipids. On the other hand, we examined the individual effects of mucins, DNA, or lipids by using these components dissolved separably in PBS.

### 2.5 Bacterial infection in the presence of epithelial cells

The 16HBE cells bearing the F508del mutation were seeded in 24-well plates (TPP®) at 2.10^5^ cells/well and incubated for 48 h at 37 °C/5% CO_2_. Bacteria at the logarithmic phase were then resuspended in MEM Glutamax without FBS or antibiotics. The cells were then infected with bacteria at a multiplicity of infection (MOI) of 1.

At the same time as the infection, CAMA was added at a final concentration of 16 µg/mL (corresponding to the MBC). The positive control for CAMA bactericidal activity was a sample without cells, while the negative control was epithelial cells without infection. The plates were incubated at 37 °C/5% CO_2_. After 2 h, the cells were lysed using PBS containing 0,1% Triton X-100 and scraped. After an overnight incubation at 37 °C, the Colony-Forming Units (CFU) were counted on LB plates.

### 2.6 Biofilm formation in the presence of epithelial cells

The 16HBE F508del cells were seeded at 2 × 10^5^ cells/well in a 24-well plate (TPP®) and grown for a week at 37 °C/5% CO_2_ to ensure optimal cell-to-cell adhesion_._ Bacteria at the stationary phase were then resuspended in MEM Glutamax devoid of FBS, antibiotics and red phenol. The cells were then infected with an MOI of 30 and incubated for 1 h at 37 °C/ 5% CO_2_. Following incubation, the wells were gently rinsed three times with PBS, to remove bacteria not attached to cells. Subsequently, MEM-Glutamax supplemented with 0.4% arginine was added to each well, and the plate was incubated overnight (16h) at 37 °C/ 5% CO_2_. To assess the efficacy of CAMA in eradicating biofilms, the medium was removed from the wells after 16 h of biofilm formation. It was then replaced with MEM Glutamax and 0,4% supplemented in CAMA (at a final concentration of 16 or 160 μg/mL) for 2 h before biofilm recovery. The bronchial cells were then lysed to recover the biofilm. This is achieved by adding concentrated 0.1% Triton X-100, incubating for 5 min at 37 °C/ 5% CO_2_ and vortexing 1min. After an overnight incubation at 37 °C, the CFU were counted on LB agar plates.

### 2.7 Killing assay

Following an incubation period to reach the exponential phase, the bacterial suspension was diluted in MHBII to achieve an OD_600__nm_ of 0,001. Subsequently, the bacteria were inoculated in the various tested media and treated with CAMA at a concentration of 16 µg/mL. Following a 2 h incubation period at 37 °C and 120 rpm, the CFU were counted. A killing assay against MRSA 7877 in PBS was used as a control.

### 2.8 Protease quantification

Protease activity was quantified using the Pierce Fluorescent Protease Assay kit (Pierce Biotechnology, Thermo Fischer Scientific, USA). Fluorescence was measured by FLUOstar OPTIMA BMGLABTECH.

### 2.9 Statistical analyses

For experiments using CFU, comparisons were made after log transformation to get normal distribution and variance homogeneity prior analyzing data. Statistical Analyses were performed using GraphPad Prism 8.0.1 software. Data were analyzed using T-test to compare 2 groups. Data were analyzed using one-way ANOVA with Bonferroni's multiple comparisons to compare more than 2 groups. The criterion for significance was *p* < 0.05.

## 3 Results

### 3.1 CF sputum inhibits the bactericidal activity of CAMA

**Fig. 1:**
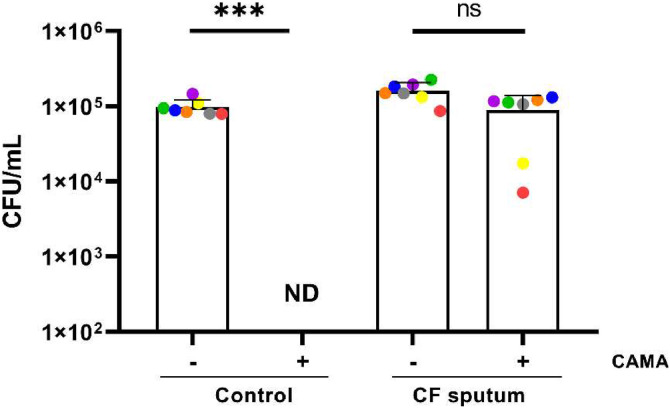
CAMA activity in CF sputum. The MRSA 7877 strain was incubated 2 h with CAMA at 16 µg/l (+) or H_2_O (-) in PBS or in CF sputum. Bactericidal effects were monitored by counting the bacterial CFU and expressed as CFU counts per milliliter. ND, not detected (< 100 CFU per milliliter). A colored dot represents the mean from at three independent experiments of the assay with a patient's sputum. *** *p* < 0.001.

Given the complex nature of CF sputum, which may interfere with CAMA activity, we sought to ascertain whether this peptide retains its bactericidal activity against an MRSA strain in CF sputum collected from seven CF patients. Compared to PBS control, no CAMA activity was observed in CF sputum (Fig. 1).

**Fig. 2:**
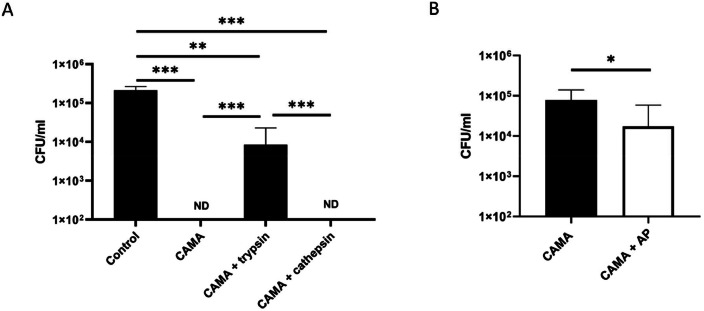
Reduction in CAMA activity in CF sputum is not only due to proteases. (A) CAMA activity in the presence of proteases. The MRSA 7877 strain was incubated 2 h with CAMA at 16 µg/l or H2O (control), and with trypsin (1 µg/ml) or cathepsin S (1 µg/ml). (B) CAMA activity in protease-free CF sputum. The activity was measured in CF sputum with a prior incubation of sputum with a cocktail of antiproteases (AP) for 30 min. Bactericidal effects were monitored by counting CFU and expressed as CFU counts per milliliter. ND, not detected (100 CFU per milliliter). Data were presented as mean±*s*.e.m. from at least three independent experiments. * *p* < 0.05, ** *p* < 0.01, *** *p* < 0.001.

Given the concentrations of proteases are known to increase in the airways of CF patients, we investigated the effects of two proteases previously detected in CF sputa (Brown et al., 2020; Reihill et al., 2020), trypsin and cathepsin S, on the bactericidal activity of CAMA against *S. aureus*. Trypsin significantly decreased CAMAs bactericidal activity against *S. aureus* after 2 h incubation (Fig. 2A). However, at the concentrations used, cathepsin S had no impact on CAMA activity. These findings prompted us to examine the real implications of proteases in the CAMA-inhibitory action of CF sputa. The seven CF sputa used in this study exhibited protease-like activities with a protease concentration of 9,7 ± 2,3 µg/ml. However, pre-incubation of CF sputa with protease inhibitors resulted in an only marginal restoration of CAMA activity (less than 1 log reduction value, Fig. 2B). Failure of protease inactivation by heating the sputa to 95 °C, to reverse totally the inhibitory effect of sputa on CAMA confirmed these results (supplementary data, figure S1). This suggests that proteases are not the only components of CF expectorations that may alter CAMA activity and that other components are involved in this alteration.

### 3.2 CAMA inhibition by CF sputum is multifactorial

**Fig. 3:**
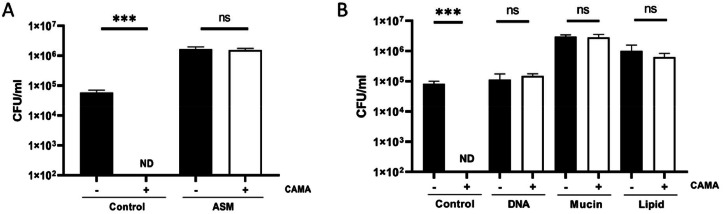
CAMA activity in the presence of sputum compounds. (A) The MRSA 7877 strain was incubated 2 h with CAMA at 16 µg/l (white bars) or H_2_O (black bars) in PBS (control) or in Artificial Sputum Medium (ASM) (A), or DNA (4 g/l), mucin (5 g/l) or lipid (0,5%) (B). Bactericidal effects were monitored by counting the CFU and expressed as CFU counts per milliliter. ND, not detected (< 100 CFU per milliliter). Data were presented as mean±s.e.m. from at least three independent experiments. ns *p* > 0.05 *** *p* < 0.001.

To explore the effect of other components of CF expectoration on CAMA activity, we examined the effects on CAMA activity of a well-established Artificial Sputum Medium (ASM). The latter, which mimics natural mucus (Sriramulu et al., 2005), is composed of DNA (4 g/l), lipids (0,5%) and mucins (5 g/l), among other components. Incubation of *S. aureus* in ASM abolished the bactericidal activity of CAMA to an extent similar to that observed in CF sputum (Fig. 3A). It should be noted that the presence of mucins and lipids in ASM, facilitated the proliferation of the MRSA strain with an increased log value of 1 (Figs. 3B).

Next, we analyzed the individual effects of DNA, lipids and mucins on the bactericidal activity of CAMA (Fig. 3B). These components were found to inhibit the bactericidal activity of CAMA against the MRSA strain. This inhibitory effect is concentration-dependent for lipids and mucins (supplementary data, figure S2). Incubation of *S. aureus* with DNA-, mucin- or lipid-free ASM resulted in CAMA activity comparable to that observed with the whole ASM (supplementary data, figure S3). This clearly showed that all ASM components participate to CAMA inhibition by ASM and that the absence of one of these components does not restore CAMA activity.

### 3.3 CAMA is active on planktonic cells but not on bacteria living in biofilm in the presence of CF bronchial epithelial cells

**Fig. 4:**
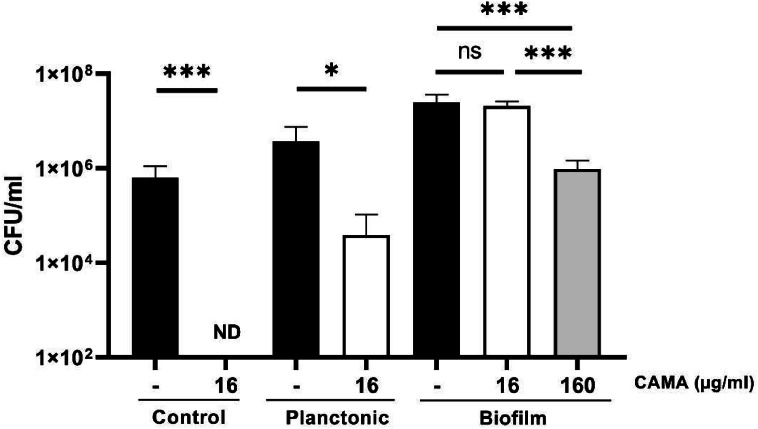
CAMA activity against the MRSA 7877 strain in the presence of CF bronchial epithelial cells. The F508del-16HBE were infected at a MOI of 1 for planktonic infection and at a MOI of 30 for biofilm infection model. For biofilm formation, non-adherent bacteria were washed after 1 h and then incubated for 16 h to allow biofilm formation. CAMA treatment was carried out at 16 or 160 µg/ml for 2 h. For biofilm infection bactericidal effects were monitored by counting the CFU and expressed as CFU counts per milliliter. ND, not detected (100 CFU per milliliter). Data were presented as mean±s.e.m. from at least three independent experiments. ns *p* > 0.05 * *p* < 0.05, *** *p* < 0.001.

To closely mimic CF airway conditions, we examined CAMA efficacy in the presence of a bronchial epithelial cell line (F508del-16HBE) carrying the F508del mutation, in both planktonic and biofilm modes to mimic the early and chronic stages of infection, respectively. In planktonic mode, when the 16-HBE cells were infected by the MRSA 7877 strain and directly treated with CAMA at 16 µg/mL, this peptide exhibited slightly diminished efficacy against the MRSA strain with a log reduction value of 2, as compared to condition in the absence of F508del-16HBE cells (Fig. 4). Similar results were observed with the methicillin-sensitive *S. aureus* SA113 laboratory strain (supplementary data, figure S5).

To mimic the chronic infections commonly observed in CF patients, a *S. aureus* biofilm model formed on F508del-16HBE cells was used after validation (*i.e.* observing biofilm formation on the CF epithelial cells with no detectable cytotoxicity in these cells, please see supplementary data, figure S4B). CAMA was unable to kill the MRSA 7877 strain at 16 µg/ml (corresponding to the MBC of CAMA), when added after biofilm formation (Fig. 4). At 10 x MIC, a weak bactericidal effect of CAMA was observed on *S. aureus* biofilm with a log reduction value of 1. Similar results were obtained with the methicillin-sensitive *S. aureus* SA113 laboratory strain (see supplementary data, figure S5).

## 4 Discussion

The majority of published studies investigating the potential therapeutic use of AMPs have examined the bactericidal effects of these peptides in conditions largely unrelated to the context of human diseases. The present work aims to investigate the bactericidal effects of a promising AMP, CAMA, in conditions that closely mimic the CF context. This AMP has already exhibited promising activity against *P. aeruginosa*, and *S. aureus*, including clinical MRSA strains, two pathogens reported to co-infect CF airways with adverse clinical outcomes (Maliniak et al., 2016; Geitani et al., 2019). Thus, CAMA represents a potential tool for treating *P. aeruginosa* and *S. aureus*.

However, CF disease is characterized by excessive sputum production in bronchial tissues, which may affect the activity of AMPs. Our findings indicate that CF sputum impairs the bactericidal efficacy of CAMA against a CF *S. aureus* strain, and this inhibition is multifactorial. While trypsin significantly reduces CAMA activity, the addition of antiproteases did not restore this total activity, suggesting that proteases are not the sole components involved in CAMA inactivation by CF sputum. Our research demonstrated that the primary components of ASM, namely DNA, mucins and lipids, can reproduce the inhibitory effects observed in natural CF sputum on the bactericidal activity of CAMA. The precise mechanisms by which DNA and mucins inhibit the bactericidal effect of CAMA on *S. aureus* remain unclear. However, it seems probable that these components bind to CAMA, thereby reducing its bactericidal activity. Indeed, previous research has indicated that DNA could reduce the activity of the AMP, LL-37 (Bucki et al., 2007). The dissolution of DNA/actin bundles and thinning of CF sputum using Dornase alfa (recombinant human DNase I) was observed to increase the amount of LL-37 peptide detected in CF sputum supernatant. This may increase the availability of this peptide for its bactericidal activity. However, in our case, the removal of DNA is not sufficient to restore the efficacy of CAMA. Previous studies have demonstrated that salivary mucins can bind to LL-37, reducing its antibacterial activity (Bucki et al., 2008; Felgentreff et al., 2006). Furthermore, ASM increased the proliferation of the MRSA strain. This is probably because ASM (especially mucins) is a source of energy for bacteria, facilitating their growth. This phenomenon is a key factor to consider because the number of bacteria determines the concentration of antibacterial molecules that must be used to eradicate the bacteria and prevent the recurrence of infection.

In the context of the therapeutic use of AMPs in CF patients, expectorations are not the only component that may alter the bactericidal activity of AMPs. The mutations in the CFTR gene result in altered ion exchange between the intra- and extracellular medium, which may cause physicochemical changes in the apical medium that alter the efficacy of AMPs. Consequently, the proliferation of *P. aeruginosa* is accelerated in the presence of CF airway cells expressing DeltaF508-CFTR compared to WT-CFTR, due to increased iron release by CF cells into the apical medium (Moreau-Marquis et al., 2008). It would therefore be of interest to test the activity of AMPs in the presence of epithelial cells bearing CFTR mutation. Using bronchial epithelial cells carrying the F508del mutation infected with *S. aureus*, we observed a slight decrease in CAMA activity against *S. aureus*, cultured in planktonic mode (close to acute infections) with CF epithelial cells. This could be explained by the binding of CAMA to bronchial epithelial cells, which may reduce its accessibility to bacteria, as previously observed with another AMP, LL-37 (Geitani et al., 2022). However, the activity of CAMA on *S. aureus* was dramatically reduced when this bacterium was cultured in biofilm mode (close to chronic infections) on CF epithelial cells. Several factors may explain this reduced activity, including a well-documented resistance mechanism of extracellular DNA (present in the matrix of biofilm) to polycationic antimicrobials, masking the negative surface charges of the bacterial membrane (Dostert et al., 2021). Therefore, CAMA does not appear to be a suitable option for treating chronic MRSA infections. This issue may be overcome by combining CAMA with a molecule exhibiting antibiofilm activity.

The present study demonstrates the ability of CF sputum components, including DNA, mucins, lipids and proteases, to influence the bactericidal activity of CAMA on MRSA strains known to infect chronically CF airways. While CAMA partially retains its bactericidal activity in the presence of bronchial epithelial cells on planktonic bacteria, it is much less effective against bacteria living in biofilm mode on these cells. Our study indicates that CF airways present a significant challenge for the effective therapeutic use of AMPs proposed to control airway bacterial infections in CF patients. Derivatives of CAMA with the amino acids in the D configuration or its encapsulation with nanoparticles could be beneficial strategies for protecting the peptide from the adverse action of CF conditions, thus facilitating its therapeutic use (Bugli et al., 2022; Sajjan et al., 2001).

## Declaration of competing interest

The authors declare the following financial interests/personal relationships which may be considered as potential competing interests: Lhousseine Touqui reports financial support was provided by Air Liquide Foundation. Lhousseine Touqui reports financial support was provided by Les motards du viaduc association. If there are other authors, they declare that they have no known competing financial interests or personal relationships that could have appeared to influence the work reported in this paper.

## Data Availability

Data will be made available on request.

## Appendix Supplementary materials

Image, application 1
