# Supplementary material for: Assessment of the efficacy of an antimicrobial peptide in the context of cystic fibrosis airways
Source: Curr Res Microb Sci. 2025 Feb 28;8:100367. doi: 10.1016/j.crmicr.2025.100367 (PMC11931299; doi:10.1016/j.crmicr.2025.100367)
Supplement: Supplementary file 1 [file mmc1.docx]

**MATERIALS AND METHODS**

**Minimal Inhibitory and Bactericidal Concentration (MIC/MBC)**

The MIC was determined by broth microdilution using 96-well plates (Corning®, USA). Following an incubation period to reach the exponential phase, the bacterial suspension was diluted in Mueller Hinton Broth II (Sigma) to achieve an OD_600nm_ of 0,001. Subsequently, the bacteria were incubated with a double dilution range of CAMA from 1 to 128 µg/ml. Plates were incubated at 37 °C for 18-20 h The MIC was defined as the lowest concentration of CAMA that inhibited visible bacterial growth. Subsequently, 10 µL of the wells that showed no visible bacterial growth were plated on an LB agar plate and incubated for 24h at 37°C. The MBC was defined as the CAMA lowest concentration where no CFU was observed.

**Validation of biofilm formation on epithelial cells**

The biofilm model was validated using the protocol described for biofilm formation in the presence of epithelial cells with some modifications to visualize biofilm by microscopy. Cells were seeded in 8-well plates (Ibidi). Before infection, the nuclei were stained with Hoechst (Thermo scientific, 1 µg/ml) and then, rinsed 3× with PBS (Gibco). After infection with the green fluorescent protein (GFP)-tagged SA113 (kindly provided by Dr Pablo Iturbe of Navarrabiomed in Pamplona, Spain), the cells were fixed with 4 % paraformaldehyde for 15 min and then rinsed 3× with PBS. The cells were then incubated for 1h with propidium iodide (Invitrogen, 10 µg/ml) to evaluate toxicity on bronchial cells. After 3 washes with PBS, the samples were observed by confocal fluorescence microscopy using Olympus FV3000. The images were processed with Imaris software V9.9 (Oxford Instruments, Abingdon-on-Thames, UK).

**RESULTS**

Table S1: MIC and MBC of S. aureus strains used in this study.

| *S. aureus* strain | MIC (µg/ml) | MBC (µg/ml) |
| --- | --- | --- |
| SA113 | 8 | 16 |
| MRSA 7877 | 8 | 16 |


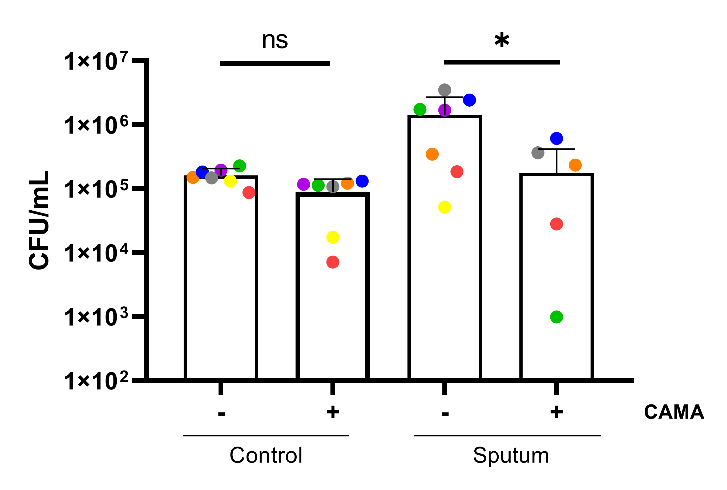


Figure S1: Inactivation of proteases by heating does not restore CAMA activity in CF sputum. The MRSA 7877 strain was incubated 2h with CAMA at 16 µg/l or H2O (control) in CF sputum pre-incubated at 95°C during 10 min. Bactericidal effects were monitored by counting CFU and expressed as CFU counts per milliliter. A colored dot represents the mean from three independent experiments with one patient's sputum. * p < 0.05


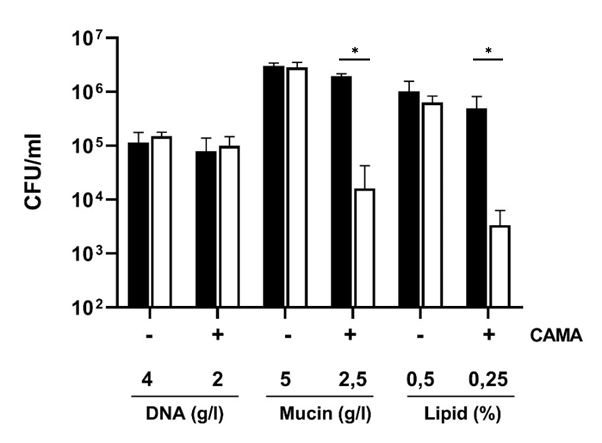


Figure S2: Influence of the ASM compounds at various concentration on CAMA activity. The MRSA 7877 strain was incubated 2h with CAMA at 16 µg/l or H_2_O in PBS with DNA, mucins or lipids at different concentrations. Bactericidal effects were monitored by counting the CFU and expressed as CFU counts per milliliter. * p < 0.05


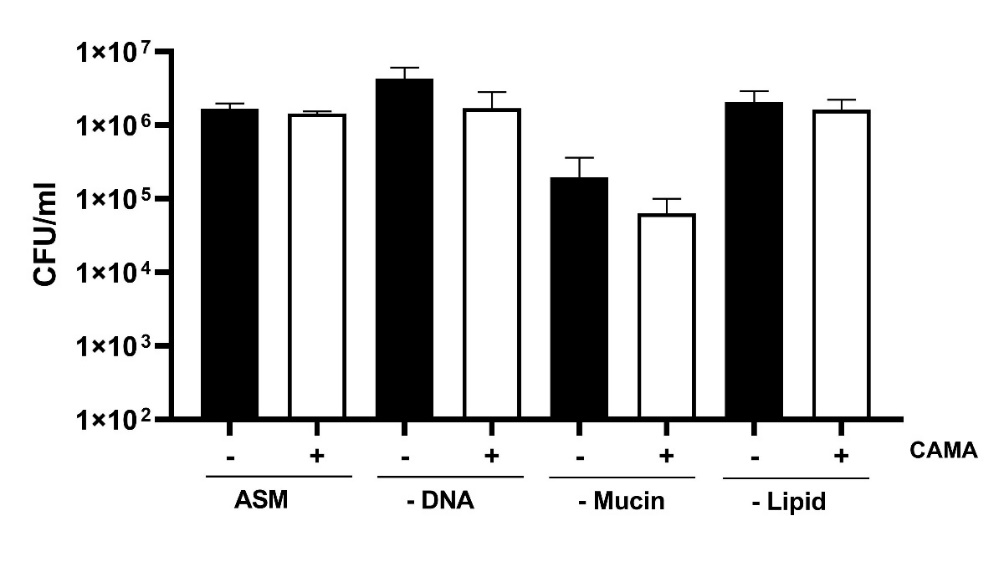


Figure S3: Impact of the absence of different ASM compounds on CAMA activity. The MRSA 7877 strain was incubated 2h with CAMA at 16 µg/l (white bars) or H_2_O (black bars) in DNA-, mucin- or lipid-free ASM. Bactericidal effects were monitored by counting the CFU and expressed as CFU counts per milliliter.


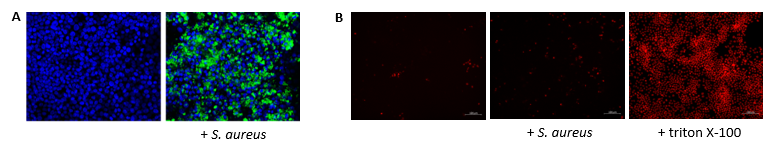


Figure S4: S. aureus biofilm formation on CF bronchial epithelial cells. The F508del-16HBE cells were cultured in plates for 7 days and then stained with Hoechst (blue). The cells were then infected at an MOI of 30 with a GFP strain of S. aureus (green). Non-adherent bacteria were washed after 1h and 16h incubation to allow biofilm development. The biofilm was then observed by confocal laser scanning microscopy (x20) (A). Cell toxicity (B) was assessed (x10) with propidium iodide labelling (red) after biofilm formation. A positive control was obtained with triton X-100.


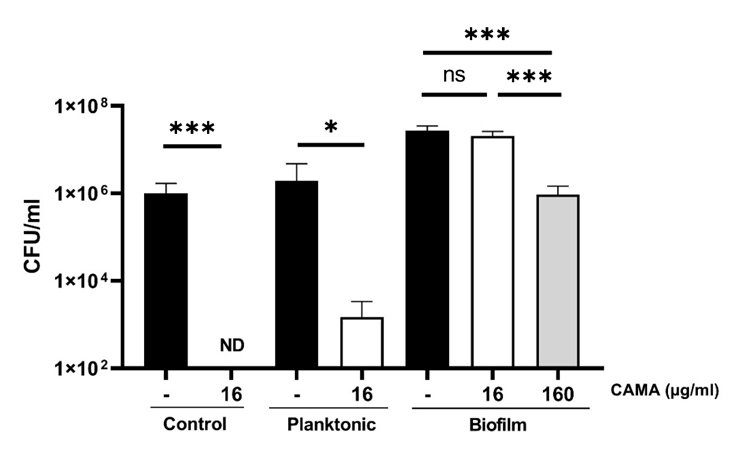


Figure S5: CAMA activity on SA113 strain in the presence of CF bronchial epithelial cells (F508del-16HBE). These cells were infected at a MOI of 1 for planktonic mode and at a MOI of 30 for biofilm mode. For biofilm formation, non-adherent bacteria were washed after 1h and then incubated for 16h to allow biofilm formation. CAMA treatment was carried out at 16 or 160 µg/ml for 2 hours. Bactericidal effects were monitored by counting the CFU and expressed as CFU counts per milliliter. ND, not detected (<100 CFU per milliliter). ns p> 0.05 * p < 0.05, *** p < 0.001
